# Validating exacerbations of asthma in electronic health records: a systematic review

**DOI:** 10.1183/16000617.0004-2026

**Published:** 2026-05-27

**Authors:** Elizabeth Moore, Zakariah Gassasse, Ian Sinha, Daniel B. Hawcutt, Jennifer K. Quint

**Affiliations:** 1 School of Public Health, Imperial College London, London, UK; 2 Institute of Life Course and Medical Sciences, Faculty of Health and Life Sciences, University of Liverpool, Liverpool, UK; 3 Alder Hey Children's Hospital, Liverpool, UK; 4 Division of Women's and Children's Health, University of Liverpool, Liverpool, UK

## Abstract

**Background:**

Previous studies have shown that the algorithms and code lists used to define asthma exacerbations vary across different sources of data, if reported at all. Defining and validating asthma exacerbations in electronic health records (EHR) would help to improve future research on asthma using EHR by leading to more consistent and comparable evidence.

**Methods:**

We systematically reviewed the literature to evaluate studies that define exacerbations of asthma in EHR and report which algorithms have the highest validity. An adapted version of the QUADAS-2 designed for this review was used to assess risk of bias.

**Results:**

Of the studies yielded by the search, only five met the inclusion criteria. Eligible studies used algorithms that contained codes from versions or modifications of either the 9th or 10th revisions of the International Statistical Classification of Diseases and Related Health (ICD-9 or ICD-10), and validity scores varied. Using the ICD-9 code 493 within algorithms to detect asthma exacerbations, sensitivity scores varied from 44.8% to 91.28% and specificity was >85%. Using the ICD-9 code 493.xx as the principal and secondary diagnosis in claims data, validity measures were all >85%. Using the ICD-10 code J45, scores for sensitivity, specificity and negative predictive value were also all >85%.

**Conclusions:**

Algorithms have been used to identify asthma exacerbations in EHR with varying degrees of validity. Algorithms including the ICD-9 code 493.xx or the ICD-10 code J45 to detect asthma exacerbations had high validity scores. However, there was a risk of bias in these studies and urgent work is needed using robust methods to validate definitions for future research using EHR.

## Shareable abstract

**Definitions of asthma exacerbations in electronic health records (EHR) using code lists vary according to different sources of data. Urgent research is required to validate and define the most accurate codes to identify asthma exacerbations in EHR.** https://bit.ly/4sKGuIQ

## Introduction

Asthma is a common chronic respiratory disease characterised by inflammation and smooth muscle constriction in the airways, resulting in symptoms such as cough, chest tightness, shortness of breath and wheeze [1]. According to the Global Initiative for Asthma (GINA), asthma exacerbations are defined as episodes of increased asthma severity that require medical intervention [2]. Exacerbations, or “attacks” as preferred by patients with asthma [3], are heterogeneous and can vary from mild symptoms requiring the use of inhalers to severe, life-threatening episodes that require urgent treatment in hospital [4]. Asthma attacks reportedly claim the lives of over 380 000 people worldwide each year [5]. In the UK, severe exacerbations have accounted for over 90 000 UK hospital admissions per annum [6]. There is also evidence of heterogeneity within different severities of asthma, and validating the criteria to define asthma exacerbations may improve treatment and patient outcomes [7].

Electronic health records (EHR) are valuable sources of data that can be used to understand diseases, treatments and outcomes, and are increasingly used to allow longer-term follow-up of clinical trials. Consisting of data collected from different settings such as primary and secondary care, EHR can provide researchers with large data samples that are more generalisable to wider populations. In the UK, the Clinical Practice Research datalink, a database of anonymised primary care records, can be used to research treatment outcomes for conditions such as asthma. Other examples of global health records databases that can be utilised for asthma research include the Danish National Patient Registry in Denmark [8] and the Epic Systems Corporation in the USA [9, 10]. Clinical coding, whether a single code or an algorithm comprising multiple codes, can be used to retrieve health records [11] from such databases. Researchers most commonly use codes from the International Statistical Classification of Diseases and Related Health Problems 9th and 10th revisions (ICD-9 and ICD-10) [12] or SNOMED CT, which are used globally in EHR databases. The 10th revision of the ICD was introduced by the World Health Organization to enhance coding of clinical information because it contains more codes than ICD-9 and an alphanumeric system [12]. The 11th revision was adopted in 2019, and the transition to implementation is currently in progress [13]. From a clinical viewpoint, it remains to be seen whether the updated revisions are superior. In the UK, diagnoses are commonly recorded as SNOMED CT codes, which are coded clinical terms used in general practice primary care databases in the National Health Service (NHS) [14, 15]. In addition, natural language processing and machine learning techniques can be used to identify asthma diagnoses in large databases through automated algorithm generation [16–18].

However, using clinical data from EHR for research is not without drawbacks, because they are not primarily designed with research in mind. The main purpose is to assist with the provision of healthcare, and in some countries such as the USA, they are designed for billing and reimbursement purposes. EHR are also real-world data (RWD), and the recording of RWD is not to clinical trial standards, with under-reporting of adverse effects and harms in RWD [19, 20]. Reliable research requires the ability to compare study findings, and validating definitions of clinical conditions is vital for this [21]. To assess the validity of the identification algorithms, studies commonly compare EHR data with a gold standard, such as chart reviews by treating physicians, patient questionnaires or linkable datasets [22]. Validation can then be measured as a positive predictive value (PPV), negative predictive value (NPV), sensitivity or specificity. Previous validation studies have found significant heterogeneity in the algorithms used to identify asthma exacerbations in EHR [23], and therefore data cannot be reliably compared. In this review, we aimed to report the methods and findings of studies that validated definitions of asthma exacerbations in EHR and to identify the most effective algorithm.

## Methods

MEDLINE and Embase were searched *via* the Ovid interface (July 2024) for the key concepts of “asthma”, “electronic health records” and “validation”. The full search strategy is described in the supplementary materials. In order to detect the validation terms, a similar search strategy was used to that of Stone *et al*. [24] and based on the search methodology by Benchimol *et al*. [25], along with strategies used in similar reviews of validation studies in EHR databases [11, 26–28]. The full methods are described in our previously published protocol [29] and the Preferred Reporting Items for Systematic Reviews and Meta-Analyses (PRISMA) guidelines were followed to report this review [30].

### Inclusion and exclusion criteria

For inclusion, studies needed to meet the following criteria: published before 30 May 2024 and written in English; data must be from an EHR or administrative claims database; validated algorithms for asthma exacerbations or attacks must be compared against a reference or gold-standard definition (*e.g.* review of records by treating physicians); and measures of validity must be included (*e.g.* PPV, NPV, sensitivity and specificity) or must be calculable from information provided in the study.

Studies were excluded if they only looked at asthma diagnosis rather than asthma attacks/exacerbations.

### Data management and synthesis

All articles were screened using Covidence systematic review software (Veritas Health Innovation, Melbourne, Australia) by two reviewers (E. Moore and Z. Gassasse). Any disagreements were resolved by consensus or arbitration with a third reviewer (J.K. Quint). Two reviewers read the full texts and independently extracted study details. Data were tabulated and stored in Microsoft Excel (Microsoft, Redmond, Washington, USA) and included the following: study details (including title, first author, year of publication), study aim/research question, EHR database used, population (location, time period), type of algorithm(s) used to detect asthma exacerbations (*e.g.* clinical coding scheme), reference/gold standard used to compare the algorithm(s) against, measure(s) of validity (*e.g.* PPV, NPV), results of validated measures, and prevalence of asthma exacerbations.

Risk of bias (ROB) was assessed using a quality assessment tool for diagnostic accuracy studies known as QUADAS-2 [31]. We adapted the QUADAS-2 to this specific review as done in other similar reviews [24, 32] using a recommended reporting checklist by Benchimol *et al*. [25] for use in validation studies of health administrative data. Our tailored QUADAS-2 can be found in the supplementary materials.

## Results

**FIGURE 1:**
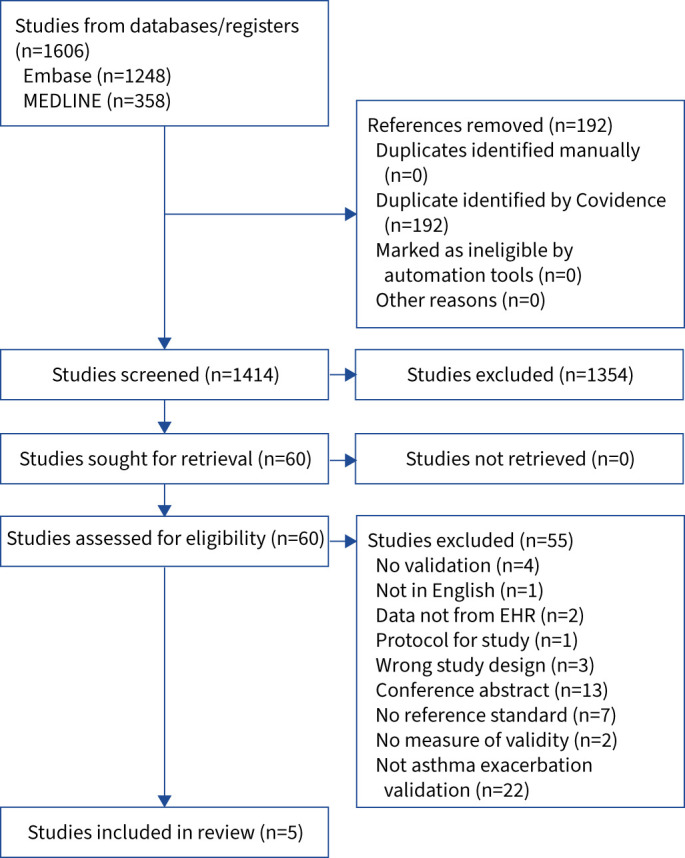
Preferred Reporting Items for Systematic Reviews and Meta-Analyses flowchart. EHR: electronic health records.

The search yielded 1414 studies (figure 1). Following title and abstract screening by two reviewers (E. Moore and Z. Gassasse), 60 articles were deemed eligible for full-text screening. Of these, only five studies met the inclusion criteria. Table 1 summarises details of these included studies, and table 2 summarises the results of validated algorithms that were used to identify asthma exacerbations. Three studies were from the US [9, 10, 16], one study examined two data sets in Australia and New Zealand [33], and one study analysed data from a patient registry in Denmark [8]. For the index tests, *i.e.* the code types to identify asthma exacerbations in the EHR, the studies used codes from ICD-9 [10], ICD-9-CM (Clinical Modification) [16], ICD-10 [8, 9] or ICD-10-AM (Australian Modification) [33]. The reference standards used to validate the algorithms were medical chart reviews conducted by clinicians in three out of five studies [8–10]. These three studies reviewed coded medical records for asthma-related emergency department (ED) visits/hospital admissions within electronic databases in which the ICD-9 or ICD-10 codes indicated that the hospital encounter was due to asthma. From the described methods, it is not clear whether the chart reviewers had all of the records, nor if the chart reviews were carried out by the primary physicians of the patients. In the study by Moth *et al*. [8], only one physician reviewed the records. For the remaining two studies, a gold-standard chart review was not used.

**TABLE 1:** Summary of included studies

Study	Country and period	Aim	Population characteristics	Data source	Code type
**McKenzie 2005 [33]**	Australia (2001–2002) and New Zealand (2003)	To determine the validity of the public submission for hospitalisations as a result of asthma and inform future changes to ICD-10-AM	Children and adults: 13% extracted from children's hospitals and 87% extracted from general hospitals	Australian hospital morbidity data from the Australian Institute of Health and Welfare; and data supplied by the New Zealand Health Information Service	ICD-10-AM
**Moth 2007 [8]**	Denmark (July and December 2002)	To explore the validity of discharge diagnoses of asthma in the Danish National Patient Registry compared with medical records	Patients 6–14 years old	Danish National Patient Registry	ICD-10
**Sanders 2007 [16]**	USA (1 November 2004–30 November 2004)	To examine the accuracy of an algorithm to identify asthma patients at triage in real-time using electronic data	Patients 2–18 years old	Computerised patient record system (StarPanel)	ICD-9-CM
**Stransky 2024 [9]**	USA (January 2017–December 2019)	To develop algorithms to identify asthma-related ED encounters and hospitalisations among patients with asthma	Patients ≥4 years old and ≤22 years old	Epic (Epic Systems Corporation) data extracted from Clinical Data Warehouse for Research	ICD-10
**Sundaresan 2018 [10]**	USA (January 2006–October 2013)	To create algorithms with high validity to identify asthma exacerbation-related ED visits	Patients 4–40 years old	EMR and claims data in software by Epic Systems Corporation	ICD-9

ED: emergency department; EMR: electronic medical records; ICD-9: International Classification of Diseases and Related Health Problems 9th Edition; ICD-9-CM: International Classification of Diseases and Related Health Problems 9th Edition Clinical Modification; ICD-10: International Classification of Diseases and Related Health Problems 10th Edition; ICD-10-AM: International Classification of Diseases and Related Health Problems 10th Edition Australian Modification.

**TABLE 2:** Asthma exacerbation definitions and validity scores

Study	Definition of asthma exacerbation	Algorithm (codes)	Reference standard	Participants (n)	PPV/derived PPV (95% CI), %	NPV/derived NPV (95% CI), %	Sensitivity/derived sensitivity (95% CI), %	Specificity/derived specificity (95% CI), %
**McKenzie 2005 [33]**	Hospital separations with a principal diagnosis of asthma, with a specific focus on the coding of “status asthmaticus” (J46), which requires documentation of “acute severe” or “refractory” asthma	ICD-10-AM code J46	Clinical terms used to describe asthma recorded by health information managers	3071 (2711 from Australia, 360 from New Zealand)	∼40	N/A	N/A	N/A
**Moth 2007 [8]**	Admission to hospital because of asthma according to DNPR with an ICD-10 code	Asthma (J45), predominantly allergic asthma (J45.0), nonallergic asthma (J45.1), mixed asthma (J45.8), asthma, unspecified (J45.9), status asthmaticus (J46.9)	Medical records reviewed by one clinician	3550	85 (80–89)	99 (99–99)	91 (87–94)	99 (98–99)
**Sanders 2007 [16]**	Presentation in ED with a symptom of asthma plus a recorded history of asthma	ICD-9 codes for presenting chief complaint: cough (786.2), dyspnoea (786.09), fever (780.6), wheezing (786.07), shortness of breath (786.05), past medical history of asthma (493)	Physician's medical notes and a summary of all orders (*e.g.* medications and laboratory tests)	154	79.3 (69.3–87.3)	69.8 (64.0–75.1)	44.8 (36.8–53.0)	91.6 (87.0–94.9)
**Stransky 2024 [9]**	Hospitalisations for asthma based on the combination of primary and secondary respiratory diagnoses plus administration of bronchodilators and systemic corticosteroids	ICD-10 codes for asthma (J45) or potentially asthma-related conditions (other respiratory disorders: J98, cough: R05, abnormalities of breathing: R06) ED encounters (two algorithms)	Chart review by two expert clinicians	Hospitalisations: 136				
		ED encounters algorithm 1: All fields: 7 rules using non-text (ICD-10 codes J45, wheezing) and text fields (chief complaint, arrival notes)		467	95.1	99.3	98.9	96.9
		ED encounters algorithm 2: Non-text fields only: 4 rules using only non-text fields		467	98.7	92.9	87.6	99.3
**Sundaresan 2018 [10]**	ED visit for which asthma was primary diagnosis	EMR: ICD-9 code of 493.xx as principal and secondary diagnosis, respiratory complaint, bronchodilator use and corticosteroids	Chart review by two reviewers	731	79.30	94.01	95.57	73.56
		Claims: principal and secondary diagnoses (ICD-9 493.xx), nebulisation codes and steroid use		731	87.93	90.86	91.28	87.36

DNPR: Danish National Patient Registry; ED: emergency department; EMR: electronic medical records; ICD-9: International Classification of Diseases and Related Health Problems 9th Edition; ICD-10: International Classification of Diseases and Related Health Problems 10th Edition; ICD-10-AM: International Classification of Diseases and Related Health Problems 10th Edition Australian Modification; N/A: not applicable; NPV: negative predictive value; PPV: positive predictive value.

In a study by McKenzie *et al*. [33], which aimed to evaluate the accuracy of clinical coding practice using ICD-10-AM and to inform future changes to the Australian Modification, the clinical terms used to describe asthma that had been recorded by health information managers were used as the reference standard. The health information managers were advised to record the clinical terms used to describe asthma in the hospital records using a standard data collection form, and these records were used in the study as the reference standard. Data from children and adults in Australia and New Zealand with a principal diagnosis of asthma were evaluated, specifically the coding of “status asthmaticus” (J46), which requires documentation of “acute severe” or “refractory” asthma. This study found that the coding of J46 had a PPV of ∼40% when compared to clinical documentation in the validation by health information managers.

Sundaresan *et al*. [10] validated a definition of asthma exacerbations using electronic medical records (EMR) and claims data of patients 4–40 years old using ICD-9 codes. An exacerbation of asthma was defined in both datasets as an ED visit for which asthma was the primary and secondary diagnosis (coded with 493.xx), along with a respiratory complaint, bronchodilator use and corticosteroid use in the EMR data, and nebulisation codes and steroid use in the claims data. The two algorithms were validated by clinician chart review, and both had a high NPV (EMR 94.01%; claims 90.86%) and high sensitivity (EMR 95.51%; claims 91.28%). The results from the claims data in this study also had high PPV (87.93%) and high specificity (87.36%). Different probability cut-offs were evaluated in this study, with the 0.5 cut-off being the most sensitive model.

Sanders *et al*. [16] carried out a feasibility study using electronic records from patients 2–18 years old, in which an asthma exacerbation was defined using ICD-9-CM codes as presentation in the ED with a symptom of asthma plus a recorded history of asthma (ICD-9-CM code 493). Physicians’ medical notes and the summary of orders (*e.g.* medications and laboratory tests) were used as the reference standard to validate the algorithm. The codes for presenting chief complaint included: cough (786.2), dyspnoea (786.09), fever (780.6), wheezing (786.07), shortness of breath (786.05) and past medical history of asthma (493). The asthma-attack identification algorithm had a sensitivity of 44.8%, a specificity of 91.6%, a PPV of 79.3% and a NPV of 69.8%.

Discharge diagnoses of children 6–14 years old from the Danish National Patient Registry were evaluated by Moth *et al*. [8]. Asthma exacerbation was defined as an admission to hospital because of asthma with one of the following ICD-10 codes: asthma (J45), predominantly allergic asthma (J45.0), nonallergic asthma (J45.1), mixed asthma (J45.8) or asthma unspecified (J45.9). Following a review of the medical records by one clinician, a sensitivity of 90%, a specificity of 99%, a PPV of 85% and an NPV of 99% were found for this algorithm.

Finally, two rule-based algorithms of ICD-10 codes were evaluated by Stransky *et al*. [9] in a US database of patients ≥4 years old and ≤22 years old with asthma-related ED encounters or hospitalisations. ICD-10 codes for asthma (J45) or potentially asthma-related conditions (other respiratory disorders: J98; cough: R05; abnormalities of breathing: R06) were included in both algorithms. In algorithm 1 for ED encounters, all fields were included, *i.e.* non-text (ICD-10 codes J45, wheezing) and text fields (chief complaint, arrival notes); in algorithm 2 for ED encounters, only non-text fields were used. Both algorithms for ED encounters were found to have high validity, with >95% for each measure of validity (PPV, NPV, sensitivity and specificity) for algorithm 1, and >85% for algorithm 2 when compared to the reference standard. For the hospitalisation algorithm, the study did not report performance metrics.

### Risk of bias assessments

**FIGURE 2:**
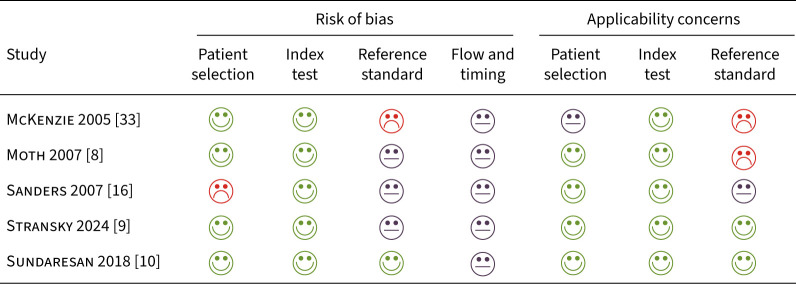
Risk of bias assessment results.

Figure 2 presents the results of the ROB assessments for each study using the adapted QUADAS-2. A high ROB was considered for McKenzie *et al.* [33] for the reference standard because these were clinical terms used to describe asthma recorded by health information managers. They may not have correctly classified asthma exacerbations, because it is unclear if they were clinicians. Furthermore, they may not have been interpreted without knowledge of the index test. Moth *et al*. [8] also had a high ROB due to applicability concerns regarding the reference standard, because the medical records were reviewed by only one clinician. It is also unclear whether the reference standard results were interpreted without knowledge of the index test in this study. The ROB for the reference standard used in Sanders *et al*. [16] was questionable because it was not clear whether the results of the reference standard were interpreted without knowledge of the index test. It was also unclear if more than one clinician reviewed the medical records. Furthermore, this study excluded low-acuity patients seen in the fast-track ED area and therefore may have been subject to selection bias. For Stransky *et al*. [9], the ROB was unclear for the reference standard because the study did not report whether the results of the reference standard were interpreted without knowledge of the index test. Finally, Sundaresan *et al*. [10] was found to have a low ROB in all domains of the adapted QUADAS-2, save for flow and timing. All the studies had unclear ROB for flow and timing, because it was difficult to determine whether all patients were included in the analysis in each study.

## Discussion

To our knowledge, this is the first systematic review evaluating methods of validating definitions for asthma exacerbations in EHR or administrative claims databases. The evidence was limited to only five studies, and we have presented the sensitivity and specificity of the ICD-9 and ICD-10 codes, with ICD-10 J45 being superior based on these publications. However, all the studies included in this review had limitations, such as small sample sizes [16] and potential ROB, particularly with regards to the reference standards. In one study, which scored highly in all validity measures for an algorithm containing the ICD-10 code J45 [8], the medical records were only reviewed by one clinician. In another, which evaluated the clinical coding of ICD-10 code J46 for status asthmaticus [33], the health information managers who recorded the reference standard may not have correctly classified asthma exacerbations. Validity measures were all >85% in a study of ICD-9 code 493.xx as the principal and secondary diagnosis in claims data [34]. This study scored low on the ROB assessment in all domains save for flow and timing.

Previous research, including a scoping review by Al Sallakh *et al*. [23], found that approaches to defining asthma or assessing asthma outcomes in EHR lacked consensus, with significant heterogeneity in the algorithms used to define asthma exacerbations. A review by Nissen *et al*. [11] also found that definitions and methods of asthma diagnosis validation vary widely across different EHR databases, and asthma symptoms present differently depending on the setting (*e.g.* primary care, secondary care and urgent care). Finally, Sharifi *et al*. [35] found a paucity of studies using rigorous methods to validate algorithms for the identification of acute asthma or bronchospasm (a hallmark of asthma) in general populations, with only three studies reporting any validation, and all were among paediatric populations.

The findings of this review are also similar to a recent systematic review validating acute exacerbations of COPD (AECOPD) [15], in which there was no clear consensus on the most sensitive and specific definition to use in EHR. As with AECOPD, asthma exacerbations can also be identified in EHR using algorithms comprising ICD-9 or ICD-10 codes. However, it was concluded that more research is needed to determine the most valid definition of AECOPD to accurately measure exacerbations in EHR. Here, we found even fewer studies aiming to validate definitions of asthma exacerbations than studies of AECOPD, highlighting an urgent need for further research in this area of asthma. Although the ICD-9 code 493.xx and the ICD-10 code J45 can detect exacerbations of asthma in EHR, more research is needed in the form of validation studies to precisely confirm which definition should be recommended for use in future research. The studies found in this review lacked clarity on whether all the patient records were included in the validations and whether the chart reviews were done by treating physicians. There is also a clear need for standardised approaches in validation methods to improve consistency of the research.

While a broad search of Embase and MEDLINE was carried out using a strategy that aimed to capture a variety of different algorithms, we acknowledge that there may be publication bias, where a detection algorithm with an undesirable validity is less likely to be published. Although many databases of EHR use the same algorithms/code lists to record clinical events, any heterogeneity in the performance of the recommended algorithm in different databases could reduce the generalisability of results. Arguably, another limitation is that only studies in English were considered eligible for our review, a pragmatic decision that was made to save time. However, only one study was excluded for this reason [36]. On further review, this study would have been excluded because there was no validation of an algorithm or code to identify exacerbations of asthma. Finally, another possible limitation and an important point to consider in future research is the location in which the study is conducted. For studies conducted in the USA [9, 10, 16], the cost of attending the ED is a potential deterrent, and the ED-based definition is only going to include those where the patient went to the ED, rather than remaining at home or being treated elsewhere. Therefore, less severe exacerbations that do not require emergency treatments are not captured in this research.

There is an indisputable case for identifying a gold-standard definition of asthma attacks/exacerbations in EHR so that future research using EHR can be robust and comparable. From a clinical perspective, the consideration of whether oral steroids and/or antibiotics are included in a gold-standard definition of an asthma exacerbation is also important, because this indicates the severity of an exacerbation. For example, these treatments will be highly relevant in clinical trial follow-up studies. Understanding how associated prescription data are included alongside clinical coding in the definition of asthma exacerbations will enhance clinical interpretations in future research.

## Conclusions

Five studies were identified using validated algorithms that comprised codes from ICD-9 and ICD-10, which can be used to identify asthma exacerbations in EHR. Although the ICD-9 code 493.xx and the ICD-10 code J45 can detect exacerbations of asthma in EHR, there was a paucity of research, and all studies that were eligible had limitations. Electronic records are enormously valuable. However, if definitions are not validated and universally agreed upon, research cannot be accurately compared between studies that utilise EHR.

**Questions for future research**

• Defining universally agreed definitions in the form of algorithms to identify medical events such as exacerbations of asthma or AECOPD within EHR through validation is of huge importance to ensure that research is accurate and comparable.

• If definitions are not validated, it leaves uncertainty in whether changes that are observed in EHR are due to clinical interventions or how the outcomes are measured and observed.

• Urgent research is needed in the form of validation studies to confirm which is the most accurate algorithm to identify asthma exacerbations in EHR.

## Data Availability

Data are available on request from the corresponding author.

## Supplementary material

**Please note:** supplementary material is not edited by the Editorial Office, and is uploaded as it has been supplied by the author. Supplementary materials ERR-0004-2026.SUPPLEMENT Supplementary material: PRISMA 2020 checklist ERR-0004-2026.SUPPLEMENT2
