# Supplementary material for: Validating exacerbations of asthma in electronic health records: a systematic review
Source: Eur Respir Rev. 2026 May 27;35(180):260004. doi: 10.1183/16000617.0004-2026 (PMC13213462; doi:10.1183/16000617.0004-2026)
Supplement: Supplementary file 1 [file ERR-0004-2026.SUPPLEMENT.pdf]

Supplementary materials

Validating the recording of exacerbations of asthma in electronic health records: a systematic review

Elizabeth Moore

Medline Search Strategy

https://ovidsp.ovid.com/ovidweb.cgi?T=JS&NEWS=N&PAGE=main&SHAREDSEARCHID=4E6O4LlyojxbaNJwo9KKTh7fuWEON7EQOtO8CZNtdWihSuE3X8XvB5puSD45nFDJC

1 exp medical records systems, computerized/ or exp electronic health records/
2 ((electronic* or online or on line or digital*) adj2 (health record* or medical record* or personal record* or patient record*)).mp.
3 ((web or internet or computer*) adj3 (health record* or medical record* or personal record* or patient record*)).mp.
4 (ehr? or phr? or ephr? or emr? or paehr?).mp.
5 (patient adj2 portal*).mp.
6 1 or 2 or 3 or 4 or 5
7 exp Asthma/
8 Respiratory Sounds/
9 Bronchial Spasm/
10 Bronchoconstriction/
11 Bronchial Hyperreactivity/
12 Respiratory Hypersensitivity/
13 (asthma* or antiasthma* or wheez* or bronchospasm or bronchoconstrict*).mp.
14 (bronch* adj3 spasm*).mp.
15 (bronch* adj3 constrict*).mp.
16 ((bronchial* or respiratory or airway* or lung*) adj3 (hypersensitive* or hyperreactive* or allerg* or insufficiency)).mp.
17 ((dust or mite*) adj3 (aller*$ or hypersensitive*)).
18 7 or 8 or 9 or 10 or 11 or 12 or 13 or 14 or 15 or 16 or 17
19 (validat* or verif*).mp.
20 (PPV or PNV or NPV or "positive predictive value*" or "negative predictive value*" or "predictive positive value*" or "predictive negative value*" or "likelihood ratio" or precision or accuracy or "receiver operating characteristic*" or ROC or kappa).mp.
21 Validation Studies/ or validation.mp.
22 validation studies as topic/ or "predictive value of tests"/ or "reproducibility of results"/
23 (valid* or reproducib* or reliab* or repeat* or eval* or predict* or responsiveness).ti,hw,kf.
24 19 or 20 or 21 or 22 or 23

25 6 and 18 and 24

Embase Search strategy

1 exp medical record/ or exp electronic health record/ or exp electronic medical record/
2 ((electronic* or online or on line or digital*) adj2 (health record* or medical record* or personal record* or patient record*)).mp.
3 ((web or internet or computer*) adj3 (health record* or medical record* or personal record* or patient record*)).mp.
4 (ehr? or phr? or ephr? or emr? or paehr?).mp.
5 (patient adj2 portal*).mp.
6 1 or 2 or 3 or 4 or 5
7 exp asthma/
8 bronchoconstriction/
9 (asthma* or antiasthma* or wheez* or bronchospasm or bronchoconstrict*).mp.
10 ((bronchial* or respiratory or airway* or lung*) adj3 (hypersensitive* or hyperreactive* or allerg* or insufficiency)).mp.
11 7 or 8 or 9 or 10
12 validation process/ or validation study/
13 (validat* or verif*).mp.
14 (sensitivity or specificity or "Sensitivity and Specificity").mp.
15 (PPV or PNV or NPV or "positive predictive value" or "predictive negative value" or "negative predictive value" or "likelihood ratio" or precision or accuracy or "receiver operating characteristic" or ROC or kappa).mp.
16 12 or 13 or 14 or 15
17 6 and 11 and 16

QUADAS-2 (adapted for validation of recording of asthma exacerbations in healthcare databases review)

Domain 1: Patient selection

A. Risk of bias

Describe methods for patient selection:

• Was a consecutive or random sample of patients enrolled? YES/NO/UNCLEAR

• Was a case-control design avoided? YES/NO/UNCLEAR

• Did the study avoid inappropriate exclusions? YES/NO/UNCLEAR

Could the patient selection have introduced bias? RISK: HIGH/LOW/UNCLEAR

(Score low if answers to all signalling questions were yes. Score high if any answers were no. Score unclear if any were answered as unclear with remainder scoring low)

B. Concerns regarding applicability

Describe included patients:

• Were patients from a single EHR database that comprised patients from one specific setting (e.g. primary or secondary care only patients)? YES/NO/ UNCLEAR

• Were patients recorded with a diagnosis of asthma?

YES/NO/UNCLEAR

Is there concern that the included patients do not match the review question?

CONCERN: HIGH/LOW/UNCLEAR

(*Score low answered yes to both questions. Score high if no to both questions. Score unclear if one marked as unclear.)*

Domain 2: Index test(s)

A. Risk of bias

Describe the index test and how it was conducted and interpreted:

• Was the detection algorithm for exacerbations of asthma designed without knowledge of the result of the reference standard (in the final validated population)? YES/NO/UNCLEAR

Could the interpretation of the index test have introduced bias?

RISK: HIGH/LOW/UNCLEAR

(Score low if all answers to signalling questions were yes. Score high if any were answered no. Score unclear if any were answered as unclear with remainder scoring low).

B. Concerns regarding applicability

• Were specific clinical codes / algorithms used to identify patients (i.e. a free text search wasn't used as part of patient identification)? YES/NO

Is there concern that the index test, its conduct or interpretation differ from the review question? CONCERN: HIGH/LOW/UNCLEAR

(Score low if clear descriptions of clinical codes/algorithms were given to identify patients.)

Domain 3: Reference standard

A. Risk of bias

Describe the reference standard and how it was conducted and interpreted:

• Is the reference standard likely to correctly classify the target condition?

YES/NO/UNCLEAR

• Were the reference standard results interpreted without knowledge of the index test?

YES/NO/UNCLEAR

Could the reference standard, its conduct, or its interpretation have introduced bias?

RISK: HIGH/LOW/UNCLEAR

(Score low if all answers to signalling questions were yes. Score high if any were answered no. Score unclear if any were answered as unclear with remainder scoring low)

B. Concerns regarding applicability

• Was there a confirmed diagnosis of asthma in the records? YES/NO/UNCLEAR

• Was diagnosis confirmed by a physician reviewing the patient's medical record?

YES/NO/UNCLEAR

• Did more than one physician review the medical record to confirm diagnosis and was

there strong agreement between the reviewing physicians? YES/NO/UNCLEAR

Is there concern that the target condition as defined by the reference standard does not match the review question? CONCERN: HIGH/LOW/UNCLEAR

(Score low if all answers to signalling questions were yes. Score high if any were answered no. Score unclear if any were answered unclear with the remainder low).

Domain 4: Flow and timing

A. Risk of bias

• Did all patients receive a reference standard? YES/NO/UNCLEAR

• Did patients receive the same reference standard? YES/NO/UNCLEAR

• Were all patients included in the analysis? YES/NO/UNCLEAR

Could the patient flow have introduced bias? RISK: HIGH/LOW/UNCLEAR

(Score low if all answers to signalling questions were yes. Score high if any answers were no. Score unclear if any were answered as unclear with the remainder scoring low).
